# Determinants of immunization status among 12- to 23-month-old children in Indonesia (2008–2013): a multilevel analysis

**DOI:** 10.1186/s12889-018-5193-3

**Published:** 2018-02-27

**Authors:** Holipah, Asri Maharani, Yoshiki Kuroda

**Affiliations:** 1 Department of Public Health, Faculty of Medicine, University of Miyazaki, 5200 Kihara, Kiyotake-cho, Miyazaki City, Miyazaki 889-1692 Japan; 2 Faculty of Medicine, Universitas Brawijaya, Malang, Indonesia; 3 Divisions of Neuroscience and Experimental Psychology, Faculty of Biology, Medicine and Health, The University of Manchester, Manchester, UK

**Keywords:** Determinants, Immunization status, Children, Indonesia, Multilevel analysis

## Abstract

**Background:**

Immunization is one of the most cost-effective public health interventions to prevent children from contracting vaccine-preventable diseases. Indonesia launched the Expanded Program for Immunization (EPI) in 1977. However, immunization coverage remains far below the United Nations International Children’s Emergency Fund (UNICEF) and World Health Organization (WHO) target of 80%. This study aims to investigate the determinants of complete immunization status among children aged 12–23 months in Indonesia.

**Methods:**

We used three waves of the Indonesian National Socioeconomic Survey (2008, 2011, and 2013) and national village censuses from the same years. Multilevel logistic regression was used to conduct the analysis.

**Results:**

The number of immunized children increased from 47.48% in 2008 to 61.83% in 2013. The presence of health professionals, having an older mother, and having more educated mothers were associated with a higher probability of a child’s receiving full immunization. Increasing the numbers of hospitals, village health posts, and health workers was positively associated with children receiving full immunization. The MOR (median odds ratio) showed that children’s likelihood of receiving complete immunization varied significantly among districts.

**Conclusions:**

Both household- and district-level determinants were found to be associated with childhood immunization status. Policy makers may take these determinants into account to increase immunization coverage in Indonesia.

## Background

Since the twentieth century, immunization and vaccination have been recognized as preventive strategies for vaccine-preventable diseases [1]. Data from World Health Organization (WHO) showed that immunization prevents 2,5 million deaths every year in the world [2]. WHO initiated the Expanded Program for Immunization (EPI) in 1974 to protect the population from six diseases: tuberculosis, diphtheria, tetanus, pertussis, measles, and poliomyelitis. This goal was considered an essential element of the WHO strategy to achieve health for all by 2000 [3]. However, in 2008, nearly 1.7 million children under five years old died of vaccine-preventable diseases, and 19.3 million children were missing six basic vaccines in 2010. More than half (53%) of those missing vaccination were in India, Nigeria, and Indonesia [4].

The government of Indonesia (GOI) showed a strong commitment to improving immunization coverage as a priority public health intervention by launching the EPI in 1977. One of the GOI’s strategies for improving immunization coverage is to bring immunization services closer to the community. It extends primary health center services from stationary and mobile health centers to community-based health services that involve non-governmental organizations (NGOs) and civil society organizations (CSOs) at the sub-village and community levels [5]. With a population of more than 220 million, Indonesia has more than 4 million children to immunize annually. The basic vaccines based on Indonesian national policy are bacille Calmette-Guérin (BCG, against tuberculosis); diphtheria, tetanus and pertussis (DTP); oral polio vaccine (OPV); measles; and hepatitis B [5]. Although immunization coverage in Indonesia is increasing year by year, it still falls short of the WHO and UNICEF’s goals as stated in their Global Immunization Vision and Strategy [3]: to reach 90% of children under the age of one with routine immunization nationwide, and at least 80% in every district in the country by the year 2020 [6]. Almost half (46.64%) of children in Indonesia did not receive complete immunization in 2012 [7].

Literature from low- and middle-income countries has shown that low income [8], lack of access to health care, high cost of health care, low parental education [9], delivery unassisted by a professional birth attendant [10], and single mothering [11] are risk factors for low immunization coverage. Identifying the factors that affect childhood immunization is essential for policy makers to establish strategies to increase immunization coverage.

Previous studies have shown that sociodemographic and geographic health factors, such as mothers’ age and education, as well as the availability of professional health attendants, affected the uptake of the first dose of measles vaccinations in Indonesia [12–14]. However, those studies were limited to measles immunizations and took only household level determinants into account. The districts’ characteristics played a crucial role in successful immunization programs in Indonesia as the country’s regions vary in terms of both geographical and economical characteristics. Furthermore, previous studies have been mostly cross-sectional and have used single-level logistic regression analysis data which failed to capture the hierarchal structure at the district level. The purpose of this study was to identify both the household- and district-level determinants of childhood immunization status in Indonesia from 2008 to 2013.

## Methods

### Study design

This study aims to identify the household- and district-level determinants of immunization status among children aged 12–23 months in Indonesia. This study used data from several sources. The 2008, 2011, and 2013 waves of the Indonesian National Socioeconomic Survey (*Survei Sosial Ekonomi* or SUSENAS) were the primary sources of household-level data. SUSENAS provided the data on child immunization status, maternal characteristics, and socioeconomic status. In addition to SUSENAS, data were sourced from national village censuses (*Potensi Desa* or PODES) from 2008, 2011, and 2013. The PODES data provided information on population and the number of health facilities in all of the villages within each district. All data were gathered by BPS (BPS-Statistics Indonesia). The aggregate data were calculated for each district. We included only health facilities that provide immunization for children, i.e. hospitals, public health centers (*Puskesmas*), and village health posts (*Posyandu*), in this study. The SUSENAS and PODES data sources were linked using district codes.

### Sampling technique

The SUSENAS data were collected using a stratified multi-stage cluster sampling with two strata (urban and rural area) for each district/municipality in SUSENAS 2008. Within each district/municipality, two-stage cluster sampling was applied to urban areas, while rural area sampling followed a three-stage cluster sampling design. In SUSENAS 2011 and 2013, sampling for both rural and urban areas used a three-stage cluster sampling design. SUSENAS 2008 included 285,904 sampled households spread across Indonesia’s provinces, while SUSENAS 2011 and 2013 included 300,000 households spread across 497 districts in Indonesia. The enumeration results data can be applied to elucidate conditions at the national, provincial and district /city levels.

PODES is a village-level census and collected various village-level (the smallest government administrative area) data. It includes information regarding infrastructure and the availability of public services in each village. The term “village” is used uniformly in referring to such units in both urban and rural areas. There were 73,198 and 77,126 villages covered in the 2008 and 2011 waves of PODES, respectively. The PODES survey was not conducted in the 2013. We thus linked SUSENAS 2013 with PODES 2011. Complete details of the methods have been published elsewhere [15–18].

### Data collection

Information about household socioeconomic status, immunization, birth attendant history, mother’s age, mother’s education and parity status was taken from SUSENAS. The data collected used questionnaires and was administered by means of face-to-face interviews. Regarding immunization information, parents were asked about the basic immunization status of their children and the number of doses of each immunization that each child had received. The data relied mostly on parents’ memory as parents were not asked to show immunization cards to the SUSENAS researchers.

Data about the number of village health posts, health centers and hospitals were taken from PODES. The PODES data were collected by means of face-to-face interviews with the head of village (the smallest government administrative area).

### Outcome variables

The participants in this study were children in Indonesia aged 12–23 months. The data on immunization status were taken from SUSENAS. The outcome variable was calculated using 12 doses of five vaccines. The definition of full immunization status was based on the national EPI schedule (Table 1). Children having reached the age of two years were excluded from this study because of the potential for confusion with booster immunizations. The numbers of children in this study were 26,219, 23,251, and 20,169 in the 2008, 2011, and 2013 waves, respectively.

**Table 1:** Schedule of routine immunization based on the Indonesian EPI schedule

Age of administration	Antigen		
0 months	BCG	HB0	OPV0
2 months	DPT1	HB1	OPV1
3 months	DPT2	HB2	OPV2
4 months	DPT3	HB3	OPV3
9 months	Measles		

### Determinants of immunization status

The determinants of immunization status were divided into household- and district-level determinants. There were eight household determinants: residence type (urban or rural), presence of a professional attendant during childbirth, mother’s employment status, mother’s age, mother’s education, parity status, wealth index and household income.

Residence type was categorized into rural and urban areas. The presence of a professional birth attendant during childbirth was defined as either not attended by a health professional or attended by a health professional (physician, midwife, or nurse). Mothers’ ages were grouped into three categories: ≤20 years old, 21–30 years old and > 30 years old. Mothers’ educational levels were defined as primary/no education, secondary, and higher than secondary. Mothers’ employment statuses were categorized as unemployed or employed. Parity status was considered low if the family had 1–2 children, medium if the children in the family numbered 3–5, and high if the family had more than five children. The wealth index was divided into five quantiles, with 1 representing the poorest quantile and 5 the wealthiest. The wealth index was derived from ownership of household such as ownership of a house or building, the type of wall, roof and floor in the home, water source and toilet facilities, and the type of electric provision. Principal component analysis was used to construct the wealth index [19]. Household income was measured using household expenditures over one year. The household expenditure variable was calculated as a log-transformed continuous variable to reduce the effect of outliers.

District-level determinants included in this study were the availability of health care providers for immunization services (hospitals, health centers, and village health posts) and the availability of health workers (doctors, nurses, and midwives). We calculated the density of the district-level determinants per 1000 population.

### Data analysis

All data were analyzed using STATA 13. Descriptive analysis was conducted to describe the profile of the participants in the analysis. Analysis was performed using a multilevel logistic regression model. The first model is a null model with no independent variables. The second model comprises household characteristics, while the third models comprises district characteristics. Considering a household *i* nested in district *j*, the model is:

$$ {E}_{ij\ast }={\beta}_0+\sum {\beta}_j{W}_j+{\beta}_{ij}{X}_{ij}+{u}_j+{\epsilon}_{ij} $$

*E*_ij*_  =  logit (*P* (*E*_ij*_  =  1)),

*W*_*j*_ is a set of district characteristics,

*X*_*ij*_ is a set of household characteristics,

*u*_*j*_ are the random intercepts varying over districts with mean zero and variance σ_u_^2^, and.

*ϵ*_*ij*_ is normally distributed with mean zero and variance σ *ϵ* ^2^.

The district characteristics in Model 3 are variables related to health facilities (hospitals, health centers, and village health posts/1000 population); doctors/1000 population and health workers/1000 population.

Our study used a multilevel approach, thus ensuring sufficient sample size at both the household and district levels. In this study, we used data from a minimum of 16 respondents in each district and 497 districts to establish a sufficient sample size at both levels [20, 21]. As Maas and Hox stated that 50 or more samples are required for level two analyses in order to ensure better analysis in the multilevel model, we believe that the findings in our study are robust [20, 21].

## Results

### Descriptive analysis

**Fig. 1:**
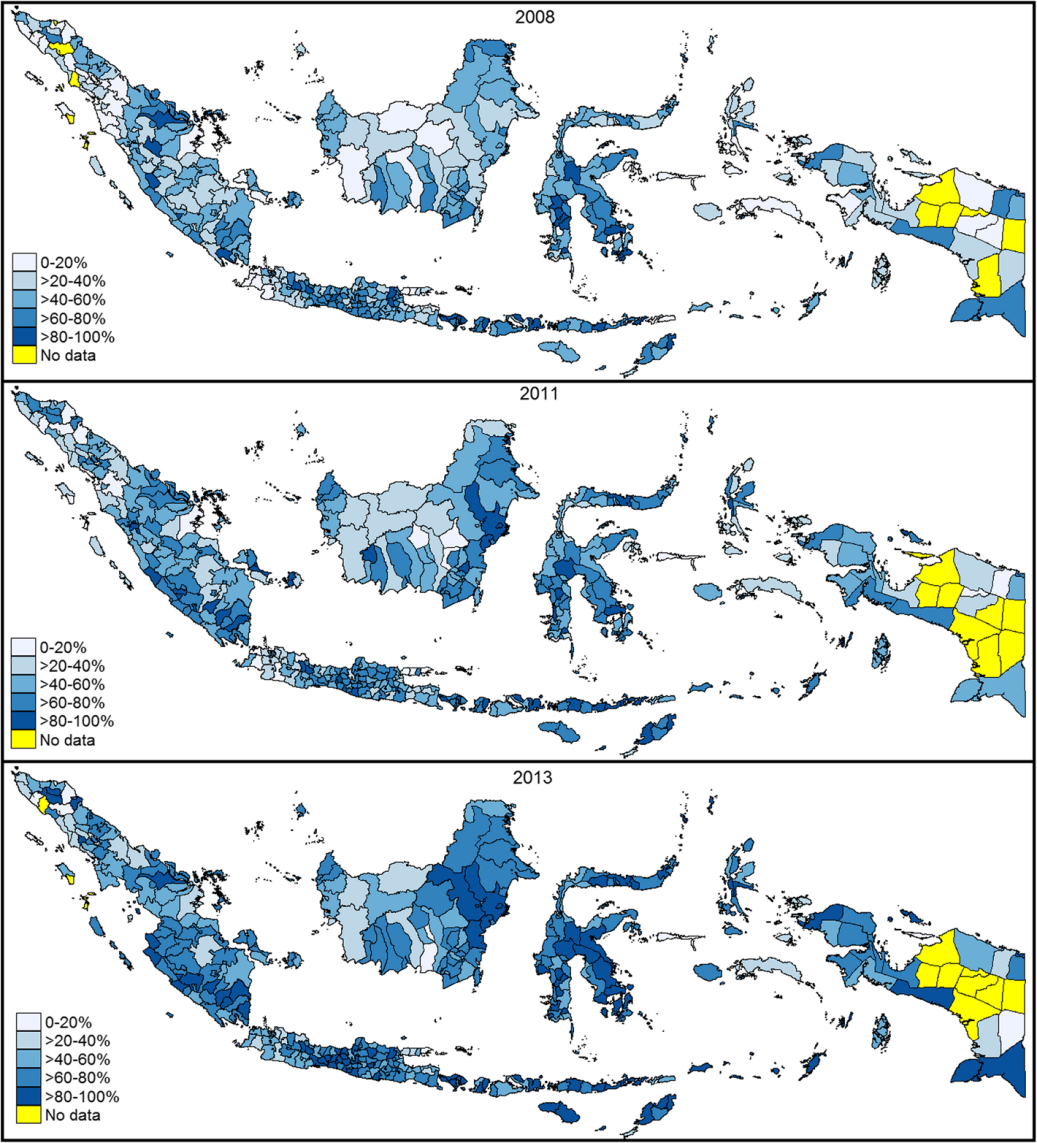
Distribution immunization coverage in Indonesia

The families of 69,639 children between 12 and 23 months of age responded to the survey in 2008, 2011, and 2013. The household and district determinants of the study participants are shown in Table 2. The proportion of immunized children increased from 47% in 2008 to 61% in 2013. However, the situation of immunization masks huge variations across districts. Figure 1 shows that more than 80% of children in 32, 57, and 116 districts received complete immunization in 2008, 2011, and 2013, respectively.

**Table 2:** Descriptive statistics on household and district characteristics

W	2008		2011		2013	
	Mean *N* (%)	SD	Mean *N* (%)	SD	Mean *N* (%)	SD
Complete immunization status						
Child received complete immunization	12,448 (47.48)		12,283 (52.83)		12,470 (61.83)	
Child missed complete immunization	13,771 (52.52)		10,968 (47.17)		7699 (38.17)	
Residential area						
Rural	17,201 (65.61)		14,228 (61.19)		11,910 (59.05)	
Urban	9018 (34.39)		9023 (38.81)		8259 (40.95)	
Birth						
Not attended by health professional	7588 (28.94)		5730 (24.64)		3625 (17.97)	
Attended by health professional	18,631(71.06)		17,521 (75.36)		16,544 (82.03)	
Mother’s age						
≤20 years	1682 (6.42)		1456 (6.26)		1260 (6.25)	
21–30 years	13,988 (53.35)		12,264 (52.75)		9812 (48.65)	
> 30 years	10,549(40.23)		9531 (40.99)		9097 (45.10)	
Mother’s education						
Primary/no education	20,950 (82.56)		13,515 (60.72)		11,151 (57.03)	
Secondary	3076 (12.12)		6196 (27.84)		5800 (29.66)	
Higher	1350 (5.32)		2546 (11.44)		2603 (13.31)	
Mother’s employment status						
Unemployed	15,144 (57.76)		13,247 (56.98)		11,617 (57.60)	
Employed	11,075 (42.24)		10,002 (43.02)		8552 (42.40)	
Parity status						
Low	15,719 (59.95)		14,563 (62.66)		12,605 (62.50)	
Medium	8879 (33.86)		7395 (31.82)		6557 (32.51)	
High	1621 (6.18)		1285 (5.52)		1007 (4,99)	
Wealth Index						
Q1 (poorest quintile)	6562 (25.03)		5566 (23.94)		4544 (22.53)	
Q2	5460 (20.82)		4843 (20.83)		4285 (21.25)	
Q3	4796 (18.29)		4607 (19.81)		3959 (19.63)	
Q4	5036 (19.21)		5698 (24.51)		4915 (24.37)	
Q5 (least poor quintile)	4365 (16.65)		2537 (10.91)		2466 (12.23)	
Household income (IDR 1000)	1961.96	1940.00	2402.49	2448.75	2926.52	3545.41
Hospitals/1000 pop.	0.02	0.02	0.03	0.03	0.03	0.03
Health centers/1000 pop.	0.27	0.2	0.27	0.19	0.26	0.19
Village health posts/1000 pop.	1.16	0.43	1.39	0.6	1.41	0.60
Doctors/1000 pop.	0.22	0.18	0.2	0.19	0.2	0.18
Health workers/1000 pop.	1.15	0.68	1.58	0.19	1.57	1.01

The majority of respondents lived in rural areas (59% in 2013), and most respondents were assisted by a health professional during childbirth. The percentage of births assisted by health professionals increased from 71% in 2008 to 82% in 2013. The majority of mothers were 21-30 years old. More than 50% of mothers had no education or completed only primary school. The numbers of employed and non-employed mothers were similar. The mean of household income increased over the period of study. Focusing on district characteristics, the densities of hospitals, health centers, doctors, and health workers remained unchanged over the five-year period. The density of village health posts increased slightly from 2008 to 2013.

Turning to the individual types of immunization covered by the study, almost 90% of children received BCG in 2008, while only half received a complete schedule of hepatitis B in the same year (see Table 3). These percentages increased to 93% and 65% in 2013. From all vaccines given in 2013, the most frequent incomplete immunization schedules received were hepatitis B and DPT at 23% and 20% respectively. Approximately 11% of children failed to receive measles and hepatitis B immunization in 2013.

**Table 3:** Percentage of children aged 12–23 months who obtained each vaccine

Type of Immunization	2008 (%)	2011 (%)	2013 (%)
DPT			
Full	15,546 (59.29)	14,830 (63.78)	14,559 (72.19)
Partial	7668 (29.25)	5925 (25.48)	4016 (19.91)
Never	3005 (11.46)	2496 (10.74)	1594 (7.90)
Polio			
Full	16,304 (62.18)	15,253 (65.6)	14,867 (73.71)
Partial	7501 (28.61)	5740 (24.69)	3762 (18.65)
Never	2414 (9.21)	2258 (9.71)	1540 (7.64)
Hepatitis B			
Full	13,681 (52.18)	13,260 (57.03)	13,308 (65.98)
Partial	8200(31.28)	6630 (28.51)	4638(23.00)
Never	4338 (16.55)	3361 (14.46)	2223 (11.02)
BCG			
Full	23,464 (89.49)	21,132 (90.89)	18,815 (93.29)
Never	2755 (10.51)	2119 (9.11)	1354 (6.71)
Measles			
Full	21,953 (83.73)	19,887 (85.53)	17,780 (88.16)
Never	4266 (16.27)	3364 (14.47)	2389 (11.84)

### Multilevel logistic regression

Table 4 shows the results of multilevel logistic regression analyses. The second model illustrates that the presence of a professional birth attendant, the mother’s age, the mother’s education level, parity status, wealth index and household income were all statistically significant (*p* < 0.01). Among household-level determinants, the presence of a professional birth attendant was the most influential factor in a child’s immunization status. Children whose births were assisted by a professional birth attendant were 1.5 times more likely to receive complete immunization than children whose births were not assisted by a professional birth attendant (OR = 1.49, 95% CI: 1.43–1.56). Children coming from high-income families were more likely to be immunized. Maternal characteristics also played an important role in children’s immunization status. Mothers who had completed a higher level of education were more likely to immunize their children than mothers who had a primary-school education or less. Younger mothers were less likely to immunize their children than older mothers. Mothers who had 3–5 children were more likely to immunize their children than those who had fewer than three children or more than five children. The relationship between mothers’ employment status and their children’s immunization status was not statistically significant. Overall, these estimates remain consistent in all three models.

**Table 4:** Determinants of children’s immunization status

Determinants	Model 1	Model 2	Model 3
Residential area			
Rural		ref	ref
Urban		1.04 (0.99–1.09)	1.04 (0.99–1.09)
Birth			
Not attended by health professional		ref	ref
Attended by health professional		1.49 (1.43–1.56)**	1.47 (1.4–1.54)**
Mother’s age			
≤20 years		ref	ref
21–30 years		1.12 (1.04–1.2)*	1.13 (1.05–1.22)*
> 30 years		1.25 (1.16–1.35)**	1.26 (1.16–1.36)**
Mother’s education			
Primary/no education		ref	ref
Secondary		1.29 (1.24–1.35)**	1.23 (1.18–1.29)**
Higher		1.30 (1.22–1.39)**	1.23 (1.15–1.32)**
Mother’s employment status			
Unemployed		ref	ref
Employed		0.98 (0.95–1.02)	0.99 (0.96–1.03)
Parity status			
Low		ref	ref
Medium		0.88 (0.84–0.92)**	0.89 (0.85–0.93)**
High		0.66 (0.61–0.72)**	0.64 (0.59–0.7)**
Wealth Index			
Q1 (poorest quintile)		Ref	
Q2		1.19 (1.13–1.26)**	1.2 (1.13–1.26)**
Q3		1.26 (1.18–1.33)**	1.27 (1.2–1.35)**
Q4		1.28 (1.2–1.37)**	1.31 (1.23–1.4)**
Q5 (least poor quintile)		1.26 (1.16–1.36)**	1.33 (1.23–1.45) **
Household income		1.3 (1.25–1.34)**	1.24 (1.2–1.29)**
District-level variables			
Hospitals/1000 pop.			1.07 (1.02–1.13)*
Health centers/1000 pop.			0.77 (0.69–0.85)**
Village health posts/1000 pop.			1.61 (1.47–1.77)**
Doctors/1000 pop.			0.87 (0.79–0.95)*
Health workers/1000 pop.			1.22 (1.15–1.3)**
Population density			
Between district variance	1.08	0.86	0.79
ICC	0.25	0.2	0.19
Median odds ratio	2.69	2.42	2.34

Note: Reported are OR (Confident interval). Sig: *P < 0.05, **P < 0.001

The third model shows that increasing the number of village health posts by one per 1000 population improved the probability of children receiving complete immunization by 60% (OR = 1.61, 95%CI: 1.47–1.77). Increasing the number of hospitals also improved the probability of children receiving complete immunization (OR = 1.07, 95% CI: 1.02–1.13). Increasing the number of health workers (midwives and nurses) by one per 1000 population increased the probability that children would receive complete immunization by 22% (OR = 1.22, 95% CI: 1.15–1.3).

As we used multilevel logistic regression models in the present research, we explain the effect between levels using median odds ratios (MOR). The MOR expresses two children from two randomly chosen districts. In the second model, for two children with the same household-level determinants, the MOR of the child living in the district with a lower propensity to vaccinate children completely is 2.42. This is a high odds ratio [22]. District-level determinants were added in the third models, and the results of the MOR decreased to 2.34; this odds ratio is still high. The propensity of children to receive complete immunization thus varies significantly between districts.

## Discussion

Using nationally representative data from Indonesia, we found that the percentage of children aged 12–23 months who received complete immunizations increased from 47% in 2008 to 61% in 2013. However, those percentages still fall short of the WHO and UNICEF’s Global Immunization Vision and Strategy goal of 80% coverage [3]. Identifying how socioeconomic factors and district characteristics affect child immunization status is thus essential to improve immunization coverage in Indonesia.

Focusing on household determinants, we found that the presence of a professional birth attendant in the delivery process had a positive and significant association with children’s immunization status. This result supports findings from previous studies linking professional birth attendance and higher immunization coverage [23–25]. In addition to assisting in the process of delivery, professional birth attendants in Indonesia provide antenatal and perinatal care, nutrition and reproductive advice, and immunization services [13, 26, 27]. Mothers who have more frequent contact with health professionals seem to be more aware of their children’s health as they receive more information about immunization and child health [1, 28]. Furthermore, based on EPI recommendations, health professionals should administer hepatitis B immunization to all newborns within 24 h of birth. Newborns can therefore receive this immunization immediately after birth if a professional birth attendant is present, and their mothers obtain adequate information from health workers about the sequence of immunizations [13, 26, 27].

Children of older mothers were more likely to be fully immunized. Women under 16 years of age in Indonesia were less likely to use any health care than older women. Younger mothers are often unable to make their own decisions; they must discuss decisions with family members. Older mothers are more likely to have experience regarding raising children and more likely to be knowledgeable about children’s health [29]. The importance of maternal education in children’s health is universally recognized. Children of more educated mothers are more likely to be fully immunized [13, 14, 30–32]. A woman with a better educational background is more likely to be aware of the importance of immunization. It is also possible that better-educated mothers are more receptive to novelty and modern ideas, more confident in making decisions for their families’ health, and more skilled at obtaining health information. Furthermore, preventive health services are more easily accepted by people with better educational backgrounds [33–36]. Women with the most education are likely to be wealthier; they also have better access to health facilities and immunization services. Education is correlated with family welfare [37].

Household income and wealth index influences the likelihood that children receive complete immunization. This result is similar to the result of many previous studies that show that children from wealthier families are more likely to be immunized than children from poorer families [1, 38–40]. Although free vaccination services are offered in Indonesia, the time and financial cost of reaching health facilities can be an obstacle to parents. Furthermore, higher wealth index are associated with better health status and health-seeking practices.

With regard to parity status, this study shows that mothers who had more than five children were less likely to immunize their children; the large number of children in the family decreased the chance of children receiving complete immunization. Previous research has shown that children in bigger families had a lower probability of receiving full immunization [41]. As the number of children in a family increases, the mother becomes busier fulfilling her children’s needs. Furthermore, a mother’s attention is divided between children if she has many children [31, 42, 43]. Children in rural areas had no significantly different probability of receiving complete immunization than children in urban areas. This may have been a result of the improvements that have been made to health facilities in rural areas of Indonesia. The GOI’s strategy for distributing health workers to rural regions has also improved rural residents’ access to health care services [5, 44].

Turning to district-level determinants, increasing the number of village health posts and health workers was related to higher immunization coverage. Among these district-level determinants, increasing the number of village health posts had the largest positive association with child immunization status. Previous research has shown that adding one village health post per 1000 population improves the probability that children will receive full immunization by 54% [7]. Village health posts (*Posyandu*) are community-based sites for health services. Under the auspices of the Ministry of Health, the Family Planning Board, and the Ministry of Internal Affairs. A village health post is a form of community empowerment, and it is managed by, from, and for the community. Community participation is the most essential factor in implementing efforts to maintain and improve basic health. Village health posts need cadres, or village health post volunteers. Cadres are voluntarily selected from among community members to organize the activities of village health posts*.* Cadres are trained at *Puskesmas* (primary health centers) by sub-district representatives of the Ministry of Health until they are able to provide the basic health care services required by their community. According to *Puskesmas* law (Indonesian Health Ministry Regulation No. 75 Year 2014), the medical doctor or midwife from the health center must help cadres master every activity. Furthermore, community leaders support *Posyandu* through fundraising and encouraging the community to actively participate in the implementation of a village health post. The community is encouraged to set up and staff the village health post once a month in every village [45]. Village health posts have a role in Indonesia’s immunization program, as the village midwife program provides immunizations as well as other maternal and child health services via the extensive village health post network. Cadres assist village midwives in distributing information and in using a personal approach to invite mothers to join the immunization program [12, 46]. The village health post program makes it easy for community members to access the immunization program. Good coordination between health centers and village health posts is an effective element in increasing the number of children who receive full immunization.

In contrast, increasing the density of health centers showed a negative association with immunization status. The most plausible explanation for this is that mothers were likely to take their children to village health posts for immunization, as shown by the positive and significant relationship between higher density of village health posts and immunization status in this study. Providing accessible immunization services is thus crucial to raising immunization coverage. The Indonesian government is committed to widening the network of primary care services that extend to the village level [47]. Health workers, including doctors, nurses, and midwives at health centers, deliver immunizations on a schedule at village health posts. Furthermore, it is easier for parents to transport their children to the village health posts given that village health posts are closer to the community than health centers, which are located in the sub-districts.

Similarly, increasing the number of hospitals is positively associated with immunization coverage. Although the GOI offers children free immunization at village health posts and health centers, increasing the number of hospitals also augments the number of children who receive full immunization. Some wealthier families choose to go directly to hospitals and pediatricians to obtain their children’s immunizations. They believe that the quality of the vaccines administered by pediatricians, who in contrast to other health workers usually use imported vaccines, may be higher quality and less likely to produce side effects.

Our study indicated that presence of higher numbers of health workers (especially midwives) influences immunization coverage, supporting the findings of previous research [48]. In addition to administering vaccines, health workers must prepare vaccines for use and share information about immunization, including immunization schedules and side effects, with patients. Health workers are also responsible for vaccine transportation and storage, thus ensuring vaccine efficacy. Higher health worker density makes a vaccine more available and broadens access for the community [48].

Increasing the number of doctors in Indonesia has a lower probability of increasing the number of children with complete immunization than increasing the number of health workers in general. Misdistribution of health workers is a problem in all developing countries, including Indonesia. In 2013, Indonesia still had not reached the WHO recommended ratio of doctors per 1000 population [49]. Inequalities in the distribution of doctors have been identified as an important policy issue for Indonesia; only 20% of doctors work in rural areas, yet they serve 70% of the population [50]. The Indonesian government introduced a policy to resolve the distribution problem by allowing nurses and midwives to prescribe essential drugs, including the administration of vaccinations, especially in rural and remote areas with few doctors [49]. In addition, health workers in the villages, especially in rural areas, accompany mothers and supervise maternal health from pregnancy through the antenatal care stage. Mothers have easier and more comfortable access to health workers than they do to doctors.

Our research found that most children in Indonesia who received partial immunizations were missing hepatitis B, measles and DPT. Hepatitis B and DPT each consist of a series of three injections, and children with partial immunizations have received only the first one or two injections. One of the possible explanations for partial immunization is the presence of immunization side effects such as fever and rashes. Another explanation may be that mothers forget to have their children immunized once the children reach a certain age, for example 9 months, the age for measles vaccination. Commonly, DPT coverage is accepted as the standard reflecting immunization program performance. The first DPT vaccine dose is an indicator of access to health care services, and the third DPT dose coverage reflects family ability to access and utilize immunization services in multiple visits [51]. The low coverage of DPT services in Indonesia shows that information regarding the need for multiple immunization visits may not reach all mothers. The increasing number of children receiving complete immunization over the years studied shows the effectiveness of the GOI’s program and its commitment to immunization. BCG vaccination was the immunization with the highest coverage. BCG vaccination is given at 0 months. The increasing number of babies delivered with the help of a health worker play a role in increasing the number of children who receive BCG vaccination.

### Policy implications

In order to improve immunization coverage, the Government of Indonesia has launched several programs. One of these is the village health post program (*Posyandu*), which provide immunization care closer to the community. Even though immunization coverage has been improving year by year, it is still below the WHO standard of 80%. Additionally, immunization coverage varies significantly between districts. This disparity might be elucidated by either household- or district-level determinants. Low maternal education levels, high poverty levels, and poor access to antenatal care and professional health attendants are among the characteristics of the districts with low immunization coverage. Those districts also have relatively few hospitals and health centers.

Our recommendations are fourfold. Firstly, the government could enhance the role of village health posts in community empowerment, especially in regions with lower immunization coverage. Improving cadres’ knowledge can be key in the distribution of information about immunization from health workers to families.

Secondly, improving mothers’ health knowledge by simply involving the community leaders would provide an approach to informing families about immunization, especially for mothers with lower levels of formal education.

Thirdly, since the increasing the number of health workers (midwives and nurses) is important for immunization coverage, the government should provide funding to increase the number of health workers at the village level. Improvement of the quality of health workers has also proven a good policy to improve quality of health. Finally, reducing economic inequality among all districts is the most important part of the solution. Reducing inequality improves wealth, educational quality, and finally health.

### Study strengths and weaknesses

There are some limitation in our study. This study is cross sectional survey, therefore, it is difficult to identify the causal mechanisms of immunization and its risk factors. Secondly, the immunization data we used was based solely on the verbal responses of parents. As they were not obliged to show immunization cards, their answers about their children’s immunization history may have been influenced by recall bias. Future data collection is needed to improve on the measurement of individual past experiences.

Despite its limitations, this study has several strengths. Firstly, we used multiyear data and a large, nationally representative sample from a population-based survey that covers every district in Indonesia. These data strongly represent Indonesian immunization coverage and its determining factors. Secondly, included in this study are not only household-level determinants, but also factors at the district level. Our study can thus capture real conditions related to immunization issues. Another strength of this study is our use of multilevel modelling analysis, which allows us to examine the clustering effect of the outcome variables.

## Conclusion

Improving the quality of education and economic conditions among the population are the most important factors in increasing immunization rates, as a component quality of health services in general. Immunization and awareness regarding immunizations are higher among mothers who delivered their babies in the presence of a professional birth attendant. Improving health access and increasing the number of health facilities and health providers in rural areas are also key to improving immunization coverage. To achieve the WHO standard on immunization coverage, as well as to improve the quality of health and health education, policy makers may establish a suitable program to encourage familiarize about immunization, particularly to targeting mothers who have little formal education.

## Abbreviations

BCG: Bacille Calmette-Guérin
BPS: BPS-Statistics Indonesia
CSOs: civil society organizations
DTP: Diphtheria, tetanus and pertussis
EPI: Expanded Program for Immunization
GOI: The government of Indonesia
HB: hepatitis B
MOR: Median odds ratio
NGOs: Non-governmental Organizations
OPV: Oral polio vaccine
PODES: Village censuses
Posyandu: Village health posts
Puskesmas: Public health centers
SUSENAS: National Socioeconomic Survey
UNICEF: United Nations International Children’s Emergency Fund
WHO: World Health Organization
